# Laboratory Evaluation of Different Insecticides against Hibiscus Mealybug,* Maconellicoccus hirsutus* (Hemiptera: Pseudococcidae)

**DOI:** 10.1155/2016/9312013

**Published:** 2016-05-22

**Authors:** Samman Fatima, Mubashar Hussain, Shama Shafqat, Muhammad Faheem Malik, Zaheer Abbas, Nadia Noureen, Noor ul Ane

**Affiliations:** ^1^Department of Zoology, Faculty of Science, University of Gujrat, Punjab 50700, Pakistan; ^2^Department of Statistics, Faculty of Science, University of Gujrat, Punjab 50700, Pakistan

## Abstract

Hibiscus mealybug,* Maconellicoccus hirsutus* (Hemiptera: Pseudococcidae), is the major pest of many vegetables, fruits, crops, and ornamental plants causing losses to the farmers and its control has been an issue of significance in the pest management. This study was aimed at evaluating different concentrations (0.06%, 0.1%, and 0.14%) of Telsta, Advantage, Talstar, Imidacloprid, and their mixtures against hibiscus mealybug in the Laboratory of Systematics and Pest Management at University of Gujrat, Pakistan. The toxic effect was evaluated in the laboratory bioassay after 24 and 48 h of the application of insecticides. The highest mortality (95.83%) was shown by Talstar and Talstar + Imidacloprid at the concentration of 0.14% after 48 h followed by Advantage + Talstar with 87.50% mortality at 0.14% concentration after 48 h of application. The study also showed that the least effective treatment observed was Advantage + Telsta with no mortality after 24 h and 25% mortality after 48 h at 0.14% concentration. The study revealed that the concentration 0.14% was highly effective in lowering the mealybug population and insecticide mixtures were effective in reducing mealybug density. The study emphasizes the use of such insecticide mixtures to develop better management strategy for mealybug populations attacking ornamental plants. However effects of such insecticide mixtures on other organisms and biological control agents should be checked under field conditions.

## 1. Introduction

Hibiscus mealybug,* M. hirsutus* (Hemiptera; Sternorrhyncha; Coccoidea; Pseudococcidae), has been one of the most devastating sap sucking pests of cultivated, noncultivated, and ornamental plants. This is an exotic pest that was first discovered in the US in Florida in 2002. It is a pest on more than 300 species in 74 plant families. Infestation of hibiscus mealybug results in malformed leaf and shoots growth and stunting and so forth. In the US yearly cost of damages caused by hibiscus mealybug and its control is about US$ 700 million, whereas global estimate is about US$ 5 billion [1]. Mealybug is represented by the largest family of scale insects with about 300 genera and 2000 species and has been reported from 35 localities of various ecological zones of the globe [2–5]. Mealybugs are phloem feeder insects which use their long and slender mouthparts to suck out fluids of plants [6]. Mealybug has a wide range of variation in morphological characters, biological adaptations, and ecological adjustability making it serious pest of almost all kinds of crops and plants. It has been recorded from several parts of Pakistan as a serious pest of cultivated and noncultivated crops and ornamental plantations [2, 7]. The pest has been reported from 183 plants in 52 families [2, 3]. Pesticides have been a large part of control for mealybug and include sodium cyanide, sulfur fumigation, chlorinated hydrocarbons like DDT and organophosphates like parathion, neonicotinoids, botanical insecticides, biosynthesis inhibitors, and insect growth regulators [8–11].

Different insecticides were evaluated against mealybug species in various parts of the world and have been found effective in reducing mealybug populations when applied at various concentrations [12, 13]. The efficacy of three insecticides, for example, Talstar (Bifenthrin 10EC), Lorsban*™* (Chlorpyrifos 50EC), and Confidor (Imidacloprid 200SL), was determined against mango mealybug (*Drosicha mangiferae)* and Lorsban was proved to be most effective for controlling mango mealybug [14].

The new chemistry insecticides are more specific for particular insects. Thus to increase crop productivity with more than one pest situation, more than one insecticide in mixtures should be used. Such mixtures can delay the development of insecticide resistance in insect pests and in this way can manage resistant population of certain insect pests [15]. The concentration of insecticides and application method have been a concern in the management strategies of mealybugs, thus requiring consistent trials for the evaluation of conventional and novel insecticides with the approach of being less hazardous against nontarget organisms and environment.

The present study was conducted in an attempt to trace out the best insecticide and most effective concentration for controlling mealybugs. They were used alone and in the form of mixtures against mealybugs. The study was conducted in laboratory conditions to determine the effect of insecticides on the management of mealybugs.

## 2. Materials and Methods

The experiment was conducted to evaluate the insecticidal activity of various commercial insecticides available in the market. The preliminary screening of selected pesticides was carried out to find out the efficacy on the control of mealybug. All the treatments were first tested at their recommended doses against adult female mealybugs but they showed very low mortality at those doses. Thus three concentrations (0.06%, 0.1%, and 0.14%) were prepared for all the treatments after reviewing the literature and efficacy was checked on these concentrations. The insecticides tested were Advantage 20 EC (Carbosulfan), Telsta 20 SL (Clothianidin), Talstar 10 EC (Bifenthrin), Imidacloprid 20 SL (Imidacloprid), and their mixtures, Advantage + Telsta, Advantage + Talstar, Advantage + Imidacloprid, Telsta + Talstar, Telsta + Imidacloprid, and Talstar + Imidacloprid, and the control (no pesticide applied) on the mortality of adult female mealybugs. The commercial insecticides were obtained from the market and their doses were prepared as per direction devised on the labels of the products.

### 2.1. Study Site

The study was conducted in May 2015 at University of Gujrat, Punjab, Pakistan (32.6367°N, 74.1674°E), with the trend of higher mealybug infestations during recent years on ornamental plantations.

### 2.2. Experimental Design

The experimental design was a Completely Randomized Design (CRD) with thirty treatments and three replications.

### 2.3. Collection of Experimental Specimens

The samples of adult females of hibiscus mealybug were collected from shoe flower plants also called China rose* (Hibiscus rosa-sinensis*) and walls surrounding the trees present at the study site. The collected specimens were identified in the Laboratory of Systematics and Pest Management, Department of Zoology, University of Gujrat.

### 2.4. Preparation of Different Concentrations of Insecticides and Insecticide Mixtures

The required volume of each insecticide was obtained by putting required quantity of formulation in the beaker and adding water to make the volume 1 liter. This procedure was repeated for all the insecticides to make concentrations of 0.06%, 0.1%, and 0.14% of all insecticides. Six different insecticide mixtures were prepared by selecting one insecticide as standard and mixing it in the other insecticides in 1 : 1 [16].

Different concentrations of insecticides were prepared by using the following method [17]:

(1) Volume  of  insecticidemL=Total  volumeL×Percentage  of  insecticide  required%Formulation  of  insecticide.

### 2.5. Laboratory Bioassay Procedure

The fresh, untreated, and noninfested host plant leaves were collected from the shoe flower plants and taken to the Systematics and Pest Management Laboratory, Department of Zoology, University of Gujrat. The experimental unit was comprised of ninety three (93) large sized Petri dishes, each containing leaves and eight adult female mealybugs. The leaves were washed thoroughly with distilled water and completely air-dried before the application of treatments. The bioassay studies were conducted based on the procedure described by Khan et al. [16] with some modifications. The treatments were applied by using Potter Tower Sprayer and experimental units were kept at room temperature 26–30°C. A slightly moistened filter paper was also placed in each Petri dish to keep the leaf material turgid during all the bioassay period.

### 2.6. Insecticide Exposure

The mealybugs were fed with fresh, noninfested host plant leaves before their release into Petri dishes. To determine the insecticidal effect of different insecticides on adult female mealybugs, the topical exposure procedure [17] was followed. This exposure method enabled controlling individual dosage [18] and prevented potential antifeedant effect of insecticides [19–21].

### 2.7. Data Collection and Statistical Analysis

The data on mortality (adults not moving when touched with a fine brush were considered as dead) was recorded after 24 and 48 h of application. Such individuals were also exposed to sunlight to confirm their death if not responding to the heat exposure. The obtained data was analyzed by using Analysis of Variance (ANOVA) and means were compared by using Least Significant Difference (LSD) Test [22]. LC_50_ and LC_90_ values for different insecticides and their mixtures were calculated by using Probit Analysis [16]. The statistical analysis was performed by using SPSS.

## 3. Results

### 3.1. Effect of Insecticides and Their Mixtures on the Mortality of Hibiscus Mealybug after 24 h of Spray

**Figure 1:**
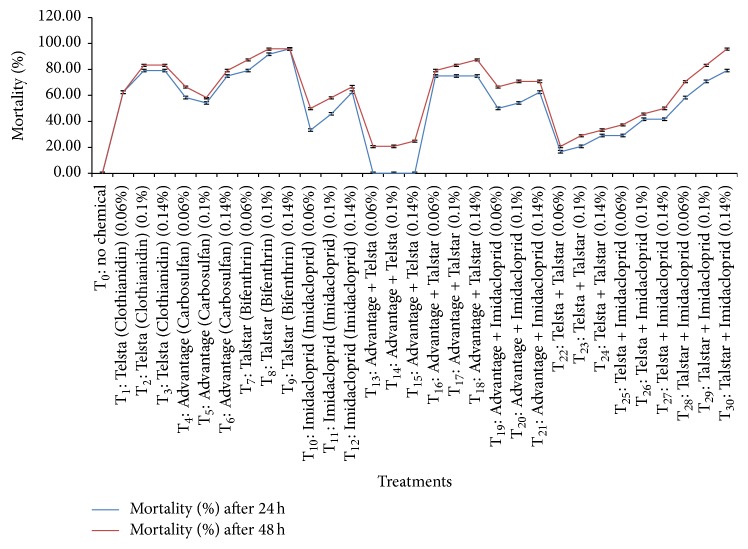
Mortality (%) of adult females of hibiscus mealybug after 24 and 48 h at different concentrations of insecticides in laboratory bioassay.

The data on mortality of adult females of hibiscus mealybug has been presented in Figure 1 that indicated significant variations in the mortality after 24 h of the application of insecticidal spray. The effect of different concentrations (0.06%, 0.1%, and 0.14%) of Advantage, Talstar, Imidacloprid, Telsta, and their combinations with three replications for each concentration was determined.

All the treatments showed significant differences with each other and also showed significant differences with the control (Table 2). There was no mortality observed in control where no insecticide was applied. The highest mortality (95.83%) was shown by T_9_ followed by T_3_ and T_30_; both of these showed 79.17% mortality. Talstar was the most effective insecticide at all concentrations after 24 h of application. The least effective insecticidal treatment (Advantage + Telsta) showed no mortality of adult female mealybugs after 24 h of spray (Figure 1). However, Advantage and Telsta when applied singly at different concentrations showed higher mortality than their mixture. The performance of insecticides in descending order showing mortality was Talstar > Talstar + Imidacloprid > Advantage + Talstar/Advantage > Advantage + Imidacloprid/Imidacloprid > Telsta + Imidacloprid > Telsta + Talstar > Advantage + Telsta at 0.14% concentration after 24 h of application in the laboratory bioassay. All the insecticide mixtures showed significant differences with each other (Table 3). Among insecticide mixtures the maximum mortality (79.17%) was shown by T_30_ (Figure 1).

### 3.2. Effect of Insecticides and Their Mixtures on the Mortality of Hibiscus Mealybug after 48 h of Spray

There were significant differences between the results of different treatments after 48 h of spray (Figure 1). All the treatments showed significant differences with respect to control, as no mortality was observed in control. The highest and similar mortality (95.83%) was observed in treatments of T_9_ and T_30_. The lowest mortality (20.83%) was observed with the application of T_13_ and T_14_. Similar trend was observed as in the results shown after 24 h of spray; in case of T_13_ and T_14_, when applied singly, they caused higher mortality than their mixture (Figure 1). Descending order for the efficacy of other treatments in causing mortality was Advantage + Talstar > Telsta > Advantage > Advantage + Imidacloprid > Imidacloprid > Telsta + Imidacloprid > Telsta + Talstar against adult female mealybugs at 0.14% concentration after 48 h of exposure to insecticides. Among insecticide mixtures the highest mortality (95.83%) was shown by T_30_ (Figure 1). However, all the insecticide mixtures showed significant differences with each other (Table 3).

### 3.3. Effect of Insecticide Concentrations and Exposure Durations on the Mortality of Hibiscus Mealybug

The insecticide concentrations and durations have significant effect on the mortality of mealybugs (Table 2). When the time of exposure of insecticides increased, mortality of mealybug also increased and vice versa (Figure 1). All the treatments also showed higher mortality with increased concentrations.

### 3.4. LC_50_ and LC_90_ for Insecticides and Their Mixtures against Hibiscus Mealybug

The insecticides with the lowest LC_50_ and LC_90_ values were considered to show highest mortality of hibiscus mealybug at lowest dose. The insecticide with the lowest LC_50_ and LC_90_ values of 0.0002% and 0.0488% calculated after 24 h of spray was Talstar (Table 1). After 48 h of spray LC_50_ and LC_90_ values for Talstar were found to be 0.000001% and 0.0032%, respectively, which were also the lowest values among all the treatments. Therefore Talstar was considered to be the most effective insecticide among all the treatments. Percentage mortality of Talstar also showed similar results (Figure 1). Among insecticide mixtures the lowest LC_50_ and LC_90_ (0.0044% and 1.3592%) were shown by Advantage + Talstar after 24 h. Thus it was observed as the most effective combination after 24 h of spray, whereas after 48 h Talstar + Imidacloprid gave the lowest LC_50_ and LC_90_ values of 0.0001% and 0.1619%, respectively, and was thus considered to be the most effective combination after 48 h of spray. Advantage + Telsta with the highest LC_50_ and LC_90_ values of 11469.15% and 6111766.00%, respectively, was considered to be the least effective treatment amongst all the treatments after 24 h of spray (Table 1). It was also considered to be the least effective treatment after 48 h of spray as it gave the highest LC_50_ and LC_90_ values of 8.0332% and 21456.76%, respectively, after 48 h. Similar results were shown by Advantage + Telsta in case of percent mortality of mealybugs (Figure 1).

## 4. Discussion

Insecticide combination Talstar + Imidacloprid is a new insecticide mixture that was not observed in the previous studies and proved to be highly effective with the lowest LC_50_ and LC_90_ values among all the insecticide mixtures against mealybug in our results. One of the possible reasons for its efficiency can be its double mode of action. Talstar is a pyrethroid and it targets “sodium channel transmission” of nervous system in insects and Imidacloprid is a neonicotinoid insecticide that acts on many types of “postsynaptic nicotinic acetylcholine receptors” present in insect central nervous system [23, 24]. Both of these insecticides are effective against sucking insects like jassids, whiteflies, aphids, and mealybugs [25, 26]. Thus the combined action of two different insecticides might be more toxic against insect pests like mealybug. This new insecticide mixture can be a better option in case of mealybug becoming resistant to already available insecticides. Thus this insecticide mixture can be helpful in “pest resistant management strategy for mealybug.”

The present study showed that Talstar was the most effective insecticide for the control of mealybugs among all the treatments when applied at different concentrations and durations of exposure. It had the lowest LC_50_ and LC_90_ values after 24 and 48 h of spray amongst all the treatments (Table 1). Similar studies were conducted by Lanjar et al. [14] and reported similar trend of mortality for Talstar. Our results differ from the results of Karar et al. [27] who evaluated Talstar against 1st instar nymphs of mango mealybugs after 24 h of spray that resulted in low mortality of 66%. In our study after 24 h of insecticide exposure, Talstar caused highest mortality but after 48 h of insecticide exposure both Talstar + Imidacloprid and Talstar caused highest mortality. These results indicated that Talstar showed higher mortality in mealybugs either used singly or used as mixture with other insecticides.

The lowest mortality of adult females of mealybug was observed with the application of Advantage + Telsta both after 24 h and after 48 h of spray. Advantage and Telsta showed higher mortality of mealybugs as compared to their mixture (Figure 1). It was also observed that Advantage + Telsta had the highest LC_50_ and LC_90_ values after 24 h and 48 h (Table 1). Thus individually both of these insecticides were more effective than their mixture. The reason for this trend of Advantage and Telsta can be due to their different modes of action. Advantage (Carbosulfan) acts by inhibiting the activity of acetylcholinesterase and Clothianidin, a novel insecticide belonging to class neonicotinoid, works as agonists on nicotinic acetylcholine receptors (nAChR) in insects [27]. Interaction of the actions performed by Advantage and Telsta might not be very effective. Therefore Advantage + Telsta caused lowest mortality of mealybugs in our results. The mixtures of selected insecticides showed significant results of mortality. The study emphasized and confirmed the results presented by Nikam et al. [28] regarding the effect of Advantage (Carbosulfan or carbyl) on mortality of mealybugs at different concentrations in laboratory bioassay while studying various insecticides when used at 0.04%, 0.05%, and 0.2% concentrations against the control of* Phenacoccus solenopsis*. Our findings are also in agreement with Karnataka et al. [29] who tested nine insecticides against the control of* P. solenopsis* and explained that Carbosulfan, profenofos, and triazofos proved comparatively better against* P. solenopsis* than other treatments.

Our results pertaining to the effect of Telsta, Advantage, and Imidacloprid are similar to those reported in a study by Pachundkar et al. [30] who evaluated nine insecticides against insect pests of cluster bean and found Telsta (Clothianidin), thiamethoxam, Imidacloprid, Acephate, Advantage (Carbosulfan), and fipronil as the most effective insecticides against the control of insect pests of cluster bean.

According to our results Advantage + Imidacloprid showed 70.83% mortality of mealybugs at 0.14% concentration after 48 h of application in laboratory bioassay. This is in accordance with the study conducted by Khan et al. [16] who studied toxicity of some insecticides alone and in combination against* Lipaphis erysimi* (mustard aphid) and reported that the best insecticidal combination was Carbosulfan + profenofos followed by Advantage + Imidacloprid, Carbosulfan + acetamiprid, and Carbosulfan + triazofos.

## 5. Conclusion

The data on mortality of mealybugs indicated effectiveness of different insecticides and their mixtures in decreasing the mealybug population. Talstar, Talstar + Imidacloprid, and Advantage + Talstar proved to be the most effective insecticide treatments for the control of mealybugs. Overall insecticides showed increasing trend in the mortality with increased concentrations. The present study suggests that Talstar, Talstar + Imidacloprid, and Advantage + Talstar are effective pesticides against mealybugs and can act as better tools in pest management for mealybug menace to crops, vegetation, and ornamental plants. The study also emphasizes checking the bioefficacy, nontarget effects, and residual toxicity of these insecticide mixtures under field conditions.

## Figures and Tables

**Table 1:** LC_50_ and LC_90 _values of different insecticides against hibiscus mealybug, *M. hirsutus,* after 24 and 48 h of application in laboratory bioassay.

Insecticides	Mortality after 24 h		Mortality after 48 h	
	LC_50_ (%)	LC_90_ (%)	LC_50_ (%)	LC_90_ (%)
Telsta (Clothianidin)	0.008	2.4575	0.0006	1.5103
Advantage (Carbosulfan)	0.0219	6.73	0.0036	9.6662
Talstar (Bifenthrin)	0.0002	0.0488	0.000001	0.0032
Imidacloprid (Imidacloprid)	0.0965	29.6223	0.0279	74.6119
Advantage + Telsta	11469.15	6111766.00	8.0332	21456.76
Advantage + Talstar	0.0044	1.3592	0.0002	0.5416
Advantage + Imidacloprid	0.0632	19.4069	0.0027	7.1883
Telsta + Talstar	3.9144	1201.158	3.7645	10054.89
Telsta + Imidacloprid	0.7204	221.0454	0.1935	516.8428
Talstar + Imidacloprid	0.0048	1.4662	0.0001	0.1619

**Table 2:** ANOVA for laboratory bioassay of different insecticidal treatments against adult females of hibiscus mealybug.

Source	Type III sum of squares	Df	Mean square	*F*	Sig.
Model	4595.789^a^	13	353.522	669.283	.000
Chemicals	672.800	9	74.756	141.526	.000
Concentration	33.100	2	16.550	31.332	.000
Duration	25.689	1	25.689	48.634	.000
Error	88.211	167	.528		
Total	4684.000	180			

^a^ *R* Squared = .981 (Adjusted *R* Squared = .980).

**Table 3:** Multiple comparisons among different insecticidal treatments against adult females of hibiscus mealybug in laboratory bioassay.

(*I*) treatments	(*J*) treatments	Mean difference (*I* − *J*)	Std. error	Sig.	95% confidence interval	
					Lower bound	Upper bound
1	2	.78^*∗*^	.242	.002	.30	1.26
	3	−1.28^*∗*^	.242	.000	−1.76	−.80
	4	1.78^*∗*^	.242	.000	1.30	2.26
	5	5.11^*∗*^	.242	.000	4.63	5.59
	6	−.33	.242	.171	−.81	.14
	7	1.00^*∗*^	.242	.000	.52	1.48
	8	4.00^*∗*^	.242	.000	3.52	4.48
	9	2.72^*∗*^	.242	.000	2.24	3.20
	10	−.11	.242	.647	−.59	.37

2	3	−2.06^*∗*^	.242	.000	−2.53	−1.58
	4	1.00^*∗*^	.242	.000	.52	1.48
	5	4.33^*∗*^	.242	.000	3.86	4.81
	6	−1.11^*∗*^	.242	.000	−1.59	−.63
	7	.22	.242	.360	−.26	.70
	8	3.22^*∗*^	.242	.000	2.74	3.70
	9	1.94^*∗*^	.242	.000	1.47	2.42
	10	−.89^*∗*^	.242	.000	−1.37	−.41

3	4	3.06^*∗*^	.242	.000	2.58	3.53
	5	6.39^*∗*^	.242	.000	5.91	6.87
	6	.94^*∗*^	.242	.000	.47	1.42
	7	2.28^*∗*^	.242	.000	1.80	2.76
	8	5.28^*∗*^	.242	.000	4.80	5.76
	9	4.00^*∗*^	.242	.000	3.52	4.48
	10	1.17^*∗*^	.242	.000	.69	1.64

4	5	3.33^*∗*^	.242	.000	2.86	3.81
	6	−2.11^*∗*^	.242	.000	−2.59	−1.63
	7	−.78^*∗*^	.242	.002	−1.26	−.30
	8	2.22^*∗*^	.242	.000	1.74	2.70
	9	.94^*∗*^	.242	.000	.47	1.42
	10	−1.89^*∗*^	.242	.000	−2.37	−1.41

5	6	−5.44^*∗*^	.242	.000	−5.92	−4.97
	7	−4.11^*∗*^	.242	.000	−4.59	−3.63
	8	−1.11^*∗*^	.242	.000	−1.59	−.63
	9	−2.39^*∗*^	.242	.000	−2.87	−1.91
	10	−5.22^*∗*^	.242	.000	−5.70	−4.74

6	7	1.33^*∗*^	.242	.000	.86	1.81
	8	4.33^*∗*^	.242	.000	3.86	4.81
	9	3.06^*∗*^	.242	.000	2.58	3.53
	10	.22	.242	.360	−.26	.70

7	8	3.00^*∗*^	.242	.000	2.52	3.48
	9	1.72^*∗*^	.242	.000	1.24	2.20
	10	−1.11^*∗*^	.242	.000	−1.59	−.63

8	9	−1.28^*∗*^	.242	.000	−1.76	−.80
	10	−4.11^*∗*^	.242	.000	−4.59	−3.63

9	10	−2.83^*∗*^	.242	.000	−3.31	−2.36

The error term is mean square (error) = .528. ^*∗*^The mean difference is significant at the 0.05 level.
